# Optimizing Clinical Assessments in Parkinson's Disease Through the Use of Wearable Sensors and Data Driven Modeling

**DOI:** 10.3389/fncom.2018.00072

**Published:** 2018-09-11

**Authors:** Ritesh A. Ramdhani, Anahita Khojandi, Oleg Shylo, Brian H. Kopell

**Affiliations:** ^1^Department of Neurology, School of Medicine, New York University, New York City, NY, United States; ^2^Department of Industrial and Systems Engineering, University of Tennessee, Knoxville, TN, United States; ^3^Department of Neurology, Icahn School of Medicine at Mount Sinai, New York City, NY, United States; ^4^Department of Neurosurgery, Icahn School of Medicine at Mount Sinai, New York City, NY, United States; ^5^Department of Psychiatry, Icahn School of Medicine at Mount Sinai, New York City, NY, United States; ^6^Department of Neuroscience, Icahn School of Medicine at Mount Sinai, New York City, NY, United States

**Keywords:** wearable sensors, Parkinson's disease, machine learning, accelerometer, gyroscope

## Abstract

The emergence of motion sensors as a tool that provides objective motor performance data on individuals afflicted with Parkinson's disease offers an opportunity to expand the horizon of clinical care for this neurodegenerative condition. Subjective clinical scales and patient based motor diaries have limited clinometric properties and produce a glimpse rather than continuous real time perspective into motor disability. Furthermore, the expansion of machine learn algorithms is yielding novel classification and probabilistic clinical models that stand to change existing treatment paradigms, refine the application of advance therapeutics, and may facilitate the development and testing of disease modifying agents for this disease. We review the use of inertial sensors and machine learning algorithms in Parkinson's disease.

## Introduction

Parkinson's disease (PD) is characterized by several cardinal motor symptoms, namely, bradykinesia, rigidity, tremor, and postural instability. As the disease progresses and higher levels of dopaminergic medications are required, the emergence of motor fluctuations and levodopa-induced dyskinesia adversely impacts the quality of life of those afflicted. The measure of disease severity dictates the management of pharmacological therapy and recommendation for advanced surgical therapies (e.g., levodopa continuous intestinal gel, deep brain stimulation). Over the past decades, numerous clinical scales have been developed to measure motor disability, usually as a snapshot in time. Many of the PD assessment scales have not been subjected to clinometric evaluation and show significant shortcomings in regards to inter-rater reliability and validity (Ramaker et al., 2002). The Unified Parkinson's disease Rating Scale (UPDRS), which has both an impairment and disability section, measures motor and non-motor aspects of the disease. Relative to other rating scales, the UPDRS and the revised version (MDS-UPDRS) have good clinometric properties (Ramaker et al., 2002; Goetz et al., 2007). However, their primary limitation rests in the subjective manner by which it is applied and the lack of continuous, real time assessment.

Sensor-based technology offers an opportunity to objectively measure motor performance in Parkinson's disease (Maetzler et al., 2016; Ossig et al., 2016). Such continuous measurements across a wide spectrum of patients with phenotypic variability stands to provide a more tailored approach to treating PD patients and improve the selection of candidates for advanced therapies such as Deep Brain Stimulation or the levodopa intestinal gel.

## Inertial sensors

Inertial sensors are constructed from accelerometers and/or gyroscopes (Zijlstra et al., 2008). Accelerometers measure the force of acceleration along a given axis but are unable to measure rotation around a vertical plane. The inclination of the sensor can be determined relative to gravity's vertical direction enabling its output to detect postural changes (e.g., sitting, standing, walking) (Mathie et al., 2004). Accelerometers operate by two components: Direct current (DC) and alternating current (AC). The DC component senses the effect of gravity and uses it to determine body position, while the AC component represents voluntary movement. Accelerometers are unable to sense rotation and therefore cannot recognize turns during walking. In addition, their measurements are subjected to spurious gravitational contributions from perturbations in the axis (Suzuki et al., 2017), which introduce a certain degree of motion inaccuracies. Gyroscopes on the other hand are able to detect the angular velocity of a rotating body and are subject to less mechanical noise. When combined with an accelerometer, turning is better evaluated with less motion dynamic artifact. However, their high-energy consumption places a significant constraint on the long-term recording.

Inertial sensor battery life is contingent on the number of sensors utilized, sampling frequency, and recording time, as well as the machine learning algorithm that is used for data processing and extraction of the signal (Habib et al., 2014). Bai et al. (2012) showed that consumption rate of the battery per hour in a triaxial accelerometer system for foreground and background execution was 2.5 and 2.25%, respectively. While reduction in the number of features and complexity of the algorithm can save on energy, the trade off could compromise accuracy. Hence, why smart phone sensors have lower resolution than external wearable dedicated sensors whose internal microcontrollers lend for more vigorous data sensing and analysis (Mehner et al., 2013).

In the following sections, we review the application of motion sensors in assessing PD motor symptoms and offer insights into various machine-learning algorithms that are the underpinnings for analyzing and potentially integrating sensor data in to clinically relevant practice paradigms. We highlight several ambulatory wearable devices—several of which (Dynaport, Physiolog, APDM, Stepwatch, Tritrac, Axivity, and Kinesia) were labeled as “recommended” from a review by Gondinho and colleagues (Godinho et al., 2016) based on their validity, reliability and sensitivity to changes in clinometric testing. A discussion of smart-phone based detection systems is beyond the scope of this review.

## Parkinson's motor symptoms

### Gait

A parkinsonian gait is described as shuffling, reduced arm swing, and multistep turning. The balance impairment and freezing that can develop increases the risk of falling and has an unpredictable response to treatment. Common assessments of gait include the Timed Up and Go test (Zampieri et al., 2010), Berg Balance Scale (Kerr et al., 2010), and the gait subscores of the UPDRS. Wearable sensors have been applied widely in analyzing gait because of its stereotyped movements and ability to be measured by single sensor positioning with three-dimensional analysis (Maetzler et al., 2016). Among PD patients, studies suggest that jerk (first derivative of acceleration) and RMS (root mean square of acceleration) measures taken from the trunk are useful and sensitive in detecting changes in balance and standing (Hubble et al., 2015) Trunk sway measurements in PD patients have also demonstrated significant deviation in angular velocity in the anterior-posterior and medial-lateral planes during stance tasks(Adkin et al., 2005). Harmonic ratio, a measure of walking stability or stride to stride variability, is based on calculations from three axes of motion. It has emerged as a sensitive parameter for differentiating PD patients from controls (Lowry et al., 2009), as well as detecting those with freezing (Weiss et al., 2015).

#### Device(s)

DynaPort MiniMod Hybrid(McRoberts, The Hague, Netherlands) contains both a triaxial accelerometer and gyroscope that is worn on the lower back. It is registered as a Class I Medical Device by the Food and Drug Administration and is utilized predominantly in an ambulatory setting. The algorithm used in this system can detect missteps during activities of daily living and has been validated to assess fall risks in PD patients (Weiss et al., 2014) based on parameters such as gait variability, consistency of gait patterns, and smoothness of gait.

Physilog (BioAGM, La Tourde-Peilz, Switzerland) attaches inertial sensors (gyroscopes and accelerometers) on various body parts (e.g., forearms, shanks, thighs, and sternum) to assess spatio-temporal parameters of gait: Stride length, stride velocity, stance double support and gait cycle (Salarian et al., 2013). In a study with PD patients with STN-DBS, significant differences were found among the gait parameters measured by this system when compared to controls, and showed high sensitivity and specificity for detecting changes in body posture (e.g., sitting, standing, walking, lying). Furthermore, a significant correlation between stride length and UPDRS gait subscore was found in this cohort (Salarian et al., 2004).

Mobility Lab System (APDM, Portland, OR) includes both ambulatory PD monitoring as well as expansive analytical software that measures outcomes from a watch size device containing six sensors that acquire 3-D linear acceleration, angular velocity and magnetic field information for directional orientation from accelerometers, gyroscopes, and magnetometers, respectively (Washabaugh et al., 2017). iSway and iTUG are two of the modules within the software system. iTUG's gait calculations, including stride length, velocity, cadence, trunk movements, turning, and turn to sit, were deemed to be most reliable and correlated well to the UPDRS III (Zampieri et al., 2010). iSWAY has also been validated to measure dynamics of postural control (Mancini et al., 2012).

The following three devices effectively measure physical activity through activity of daily living in order to determine overall gait pattern changes. Stepwatch 3 is an ankle accelerometer placed on the leg that counts strides. Its monitoring activity has been shown to be valid and reliable in detecting changes in ambulatory activity (Cavanaugh et al., 2012). Tritract RT3 (Hale et al., 2008) is a triaxial accelerometer that is worn on the lower back and tracks physical activity in 1-min epochs for up to 21 days. The sensor was found to reliably distinguish PD patients with different levels of mobility when compared to a recall questionnaire. Along the same line, the Axivity (AX3) triaxial accelerometer, which can record movement for up to 21 consecutive days, was shown to assess fall risk among PD patients based on changes in walking patterns (Godinho et al., 2016).

### Tremor

Tremor analysis delineates a tremor by frequency and amplitude. Tremor amplitude has been demonstrated to produce the greatest degree of disability and its displacement along a linear or angular axis can be differentiated with accelerometers or gyroscopes (Hess and Pullman, 2012). The addition of EMG to the analysis can further elaborate on the muscle groups recruited or synchronized during tremor generation providing a useful, quantifiable method for distinguishing various tremor subtypes. In general, wearable sensors usually employ an accelerometer encased in a miniaturized device such as a smart watch or phone app to capture the presence of tremor, but do not provide diagnostically relevant information on the nature of the tremor. Tremor algorithms tend to rely on power spectral analysis across a frequency range, (Giuffrida et al., 2009) usually in the 3-8Hz domain.

#### Device(s)

The Kinesia (Great Lake Neurotechnologies, Cleveland, OH) sensor incorporates both accelerometers and gyroscopes in a small compact sensor device worn on a finger. The data captured on PD patients assess rest, postural and kinetic tremor. The quantitative kinematic data was highly correlated with the UPDRS scores for all aspects of tremor (Giuffrida et al., 2009).

Physilog (BioAGM, La Tourde-Peilz, Switzerland) sensor achieved large sensitivity and specificity (99.5 and 94.2%, respectively) for detecting tremor. There was also a high correlation between the estimated tremor amplitude and the UPDRS rest and action tremor subscores (Salarian et al., 2007b).

### Bradykinesia

Bradykinesia, or the slow execution of movement (Marsden, 1989) manifests as a decrementation in the amplitude of repetitive movement. Assessment of bradykinesia has relied on the UPDRS, which is capable of demonstrating clinical changes, but may not be sensitive enough to parcellate the kinematic changes underlying the reduction in movement. Motion sensors offer objective measures of bradykinesia based on calculating the mean acceleration of movement from either a uniaxial or bi-axial accelerometer. A 1 to 3.5 Hz signal range (Dunnewold et al., 1997) is used in these measurements as this is the bandwidth where voluntary movements usually occur. Coexistent tremor does not affect the measurement of these movements.

#### Device(s)

The kinesia sensor (Great Lake Neurotechnologies, Cleveland, OH) was shown (Mera et al., 2012) to quantify the changes in speed, amplitude and rhythm of distal motor impairment in PD patients. Compared to UPDRS III bradykinesia scale, the sensor was more precise in evaluating the differences in amplitude and speed, which underscores fatigability or decrementation of movement.

The Physilog (BioAGM, La Tourde-Peilz, Switzerland) system demonstrated a high correlation between UPDRS hand subscores (e.g., finger tapping, hand movements and rapid alternating movements of the hand) and estimated bradykinesia measurement. This correlation persisted even with an assessment window size as short as 5-min (Salarian et al., 2007b).

### Motor fluctuations and dyskinesia

With long-term exposure of levodopa, patients can develop disabling motor fluctuations as the benefit of their levodopa wears off from one dose to another (Nutt, 1995). A subset of patients can have diphasic dyskinesia with dystonia or predictable stereotypies emerging at the beginning and end of their levodopa dose (Fox and Lang, 2008). The nature of motor fluctuations is garnered from paper-based diaries such as the Hauser diary (Hauser et al., 2006). These patient direct self-reporting systems are often subject to recall bias and noncompliance.

The use of wearable sensors to better assess motor fluctuations continues to grow and be refined. Motion sensors largely utilizing accelerometers have shown an accuracy of 70-90% to measuring fluctuations (Hoff et al., 2004; Keijsers et al., 2006; Griffiths et al., 2012; Pulliam et al., 2017), Dyskinetic movements are represented in low-frequency range often <3 Hz (Keijsers et al., 2003; Pulliam et al., 2014) and can be assessed by both accelerometers and gyroscopes with studies reporting accuracies close to 90% (Tsipouras et al., 2010, 2011). However, the quality of the dyskinesia (e.g., location, phenotypic variability including dystonic components) is not differentiated by these sensors.

#### Device(s)

The Parkinson's Kinetigraph (PKG, Global Kinetics Corporation, Melbourne, Australia) is a wrist-worn logger that utilizes an accelerometer to measure the spectral power profile of low frequencies related to bradykinesia. Using a fuzzy logic based algorithm, the sensor captures bradykinesia and dyskinesia scores in 2-min epochs over 10 days. Both the bradykinesia and dyskinesia scores are highly correlated with the UPDRS III and AIMS (Griffiths et al., 2012). Motor fluctuations were distinguished by summing the interquartile range of bradykinesia and dyskinesia scores into an objective fluctuator score (Horne et al., 2015). This score was able to distinguish fluctuators from non fluctuators with a sensitivity of 97% and a specificity of 88%.

The KinetiSense motion system (Great Lake Neurotechnologies) consists of sensors with triaxial gyroscopes and accelerometers worn on three body parts. The device's dyskinesia measurements demonstrated a high correlation with the AIMS scale as well as 80% predictive value for the algorithm (Pulliam et al., 2017).

## Machine learning algorithms

Machine learning (ML) algorithms, such as decision trees (DTs), random forest (RF), support vector machines (SVM), logistic regression (LR), naïve Bayes, hidden Markov models (HMMs), neural networks (NNs), clustering algorithms, etc. have been successfully applied in prediction and classification in medicine (Tripoliti et al., 2013; Holzinger, 2016). In the recent years, researchers have started exploring the possibility of using these algorithms to improve upon the assessment and management of PD patients (Eskofier et al., 2013; Tripoliti et al., 2013; Miljkovic et al., 2016). The use of ML algorithms in sensor based PD motor assessment is particularly important and promising due to its ability to expand traditional statistical methods into high dimensionality and nonlinear space for the volume data generated (Kubota et al., 2016)

Machine learning algorithms can generally be categorized into two classes, namely, supervised and unsupervised algorithms. Supervised learning algorithms require ground truth (e.g., direct measurements or observations) for the data used in training and testing. That is, given a set of input features and labeled response variables, the algorithms can “learn” the hidden patterns that associate the set of inputs to the responses and generalize these learning's to new observations. In ML, this refers to making a “prediction” for the new observation. Unsupervised learning algorithms, however, do not require ground truth. Instead, they are typically used to group the data into clusters with respect to their similarities.

In this section, we introduce some of the more popular machine learning algorithms. These algorithms are in general versatile and can be used to improve care and guide treatment planning with respect of an array of PD patient symptoms. Here, we highlight some of their recent applications in assessing PD motor symptoms.

## Decision trees and random forest algorithms

The DT algorithm is a machine learning technique that can be applied both for classification and regression analyses (Gordon et al., 1984). During the training stage, a collection of training data samples guides the construction of the tree-like structure. At the beginning, all the data is assigned to the root node. At each node of the tree, the algorithm generates a decision rule that splits the data samples into a number of subgroups, and one child node is created for each subgroup. Typically, the decision rule uses a single feature for the split –if the gait score is above a certain threshold, assign the DBS patient to low-frequency stimulation group, otherwise assign the DBS patient to high-frequency stimulation group. The choice of the splitting feature and the decision rule is based on some statistical criterion, such as the information gain, or gini impurity (Mitchell, 1997). If used for classification, the leaf nodes are labeled using the majority label of the data that they contain. When classifying a new data point, the algorithm passes the data point through the decision tree until it reaches the leaf node, and the label of the final leaf node is used as the prediction. Due to similarity with human decision making, the algorithm is easy to understand for practitioners (Goldman et al., 1982) DT algorithms have been effectively applied for the general classification and evaluation of movement activities collected from wearable sensors (Preece et al., 2009), which can provide an assessment of treatments for PD patients. An automatic UPDRS scoring system that uses wearable wrist-watch-type sensor measurements was developed (Jeon et al., 2017), in which the authors compared a few ML algorithms. The raw sensor data were parsed using standard signal processing techniques (Oppenheim and Schafer, 1989) to generate input features (e.g., mean amplitude, mean frequency, signal power, etc.) In this study, DT outperformed other algorithms in automatic scoring of Parkinson's tremor severity and achieved an accuracy of 85.5% compared to the tremor severity UPDRS scores provided by the professional neurologists.

Although simple to use and interpret, the DT algorithm is sensitive to the idiosyncrasies of the training data. To improve the robustness, the RF algorithm (Breiman, 2001) constructs a collection of trees by randomizing a choice of splitting features in addition to using different subsets of the training data for each tree. To generate a new prediction, the ensemble of decision trees combines the predictions of the individual trees (using the majority rule). The RF model typically improves the quality of the predictions over the single decision tree, but the results are not as easy to interpret.

RF models have been successfully applied in PD. For instance, these models have been used to predict the optimal stimulation frequency after the DBS implantation surgery based on the Unified Parkinson's disease Rating Scale (UPDRS III) scores collected at or before surgery (Khojandi et al., 2017) These models have also shown promise when used on the data collected from wearable sensors. Kuhner et al. (2017) captured 3D inertial measurements from a motion capture suit consisting of magnetometers, accelerometers, and gyroscopes in PD patients with DBS switched-off or -on, as well as healthy controls. Utilizing an RF model with probability distribution on data derived from various clinical tasks, they demonstrated the ability to detect PD patients off DBS from healthy subjects with high sensitivity and specificity. Tripoliti et al. (2013) used DT and RF models to predict the freezing of gait events in patients suffering from PD based on the information from the wearable sensors. The raw data were preprocessed to extract the entropy in the signals corresponding to the freezing of gate episodes alongside with the data from the symptom-free episodes. The authors reported 96% prediction accuracy when detecting the freezing of gate events. The decision trees were also effectively applied for the general classification and evaluation of movement activities (Preece et al., 2009) as well as resting tremor (Rigas et al., 2009). The built-in smartphone sensors were used (Arora et al., 2015) to provide a phone application for monitoring the symptoms of the PD using voice, posture, gait, finger tapping, and response times. The classification model based on RF provided 96% accuracy in predicting the motor scores of the UPDRS. (Mazilu et al., 2012) developed an RF prediction model for the freezing of gait by utilizing signals from a smartphone and wearable accelerometers with a reported accuracy of 98%.

## Support vector machine algorithm

SVM is another popular machine learning technique that is among the most successful tools for classification (Cortes and Vapnik, 1995). The SVM learning algorithm maps predictor variables to high-dimensional spaces, where the data is separated by finding a hyperplane that maximizes the “gap” between different data classes. In case of binary classification tasks, a new data point ends up either on one side of the separating hyperplane or another, generating the corresponding predictions. In (Eskofier et al., 2013) the authors collected the sensor data on the gait features of subjects walking on a treadmill. The SVM model provided a classification of the elderly vs. young walkers with 95% accuracy. The SVM algorithm was employed to classify Hoehn and Yahr stages and motor impairment levels (UPDRS-III) based on inputs from the wearable sensors (Klucken et al., 2013). Abnormalities of gait in Parkinson's patients were also detected using the SVM model with inputs from the wireless inertial sensors (Tien et al., 2010).

## Logistic regression

Logistic regression can be viewed as an extension of the linear regression to the data with categorical response variables (Agresti, 1990). The logistic regression function maps data records to the interval [0,1], so the response variables represent the likelihood of belonging to each of the target classes. Logistic regression was employed to classify movements (e.g., walking, standing, sitting) of the PD patients with the goal of providing objective information about the clinical outcomes (Albert et al., 2012). The subjects performed predefined set of activities to generate input signals and labels, while the raw accelerometer signals were split into 10-s segments labeled by the corresponding activity. A classification tool based on an LR model was designed by Salarian et al. (2007a) to classify the daily activities (posture transitions) of PD patients with DBS implants (e.g., walking, standing, sitting, and lying). The feature extraction and noise removal from the raw gyroscope signals was performed using the digital filter algorithms. Yokoe et al. (2009) used an LR model to develop an automatic finger tapping test system for PD patients to provide objective classification instead of relying on the possibly subjective visual evaluations by neurologists. The input data was obtained by observing finger tapping activities performed by PD patients and recording different measures of their durations and rhythm.

## Bayesian network classifiers

Bayesian networks (Koller and Friedman, 2009), or multivariate Gaussian classifiers, are a type of probabilistic graphical models that represent probability distributions. Naïve Bayes classifiers, a special case of Bayesian networks, are a family of probabilistic classifiers that rely on Bayes' theorem and independence assumption to stratify inputs into output classes. Specifically, they make a relatively strong assumption that input features are conditionally independent given the output's class. Despite this assumption, Naïve Bayes classifiers have shown great potential in classification tasks in various domains such as medicine and engineering, particularly when the features are weakly relevant (Hand and Yu, 2001). In PD domain, Tripoliti et al. (2013) apply Naïve Bayes to the data collected from wearable accelerometers and gyroscopes sensors, after preprocessing and feature extraction, to detect freezing of gait events. For each sensor, the authors extract entropy from each of the three axes using sliding windows. In this study, the Naïve Bayes algorithm can detect freezing of gate events with approximately 92% accuracy Other examples of Bayesian networks used in classification/prediction tasks are HMMs, which assume that the evolution of the process is Markovian where the states are unobserved (or hidden) (Koller and Friedman, 2009). For instance, (Rigas et al., 2009) use HMMs on the data collected from wearable sensors, after preprocessing and feature extraction, to accurately classify tremor severity and their type, if present, as resting vs. postural. The authors use a band-pass finite-impulse-response (FIR) filter and a low-pass FIR filter to extract the signal corresponding to tremor-induced and non-tremor-induced movements, respectively, allowing for the differentiation of tremor from other PD symptoms. Next, they use sliding windows on the processed signals and extract features such as mechanical energy of the motion, entropy, dominant frequency, and energy on dominant frequency, etc. for tremor recognition and features such as average acceleration energy of the low-frequency signal, the angles between pairs of sensors, and the angles between each sensor and the three reference axes for action/posture recognition. The developed models can quantify tremor severity with 87% accuracy and are able to discriminate resting tremor from postural tremor, and tremor from other PD motor symptoms during daily activities.

## Neural networks

NNs (Hassoun, 1995) are powerful computational models comprised of interconnected information processing units, typically referred to as neurons. NNs can be used to relate a set of input parameter to outputs through a highly nonlinear relationship. Deep learning (DL) (LeCun et al., 2015), which generally refers to the use of many layers of neurons, have recently gained widespread attention, mainly due to their superior performance in pattern and image recognition tasks. In the recent years, NN and DL have been used in various detection tasks in PD. For instance, (Das, 2010) uses NN to analyze the voice of healthy subjects and PD patients to accurately identify PD patients. The authors use a dataset of a range of biomedical voice measurements from the subjects and patients and achieve a classification accuracy of approximately 93%. In another study, DL, particularly, a convolutional NN (CNN), is used to detect bradykinesia based on the data collected from wearable sensors (Eskofier et al., 2013). As the input, the authors use three one-dimensional vectors based on the data collected from three axes of the accelerometer sensor. The network architecture includes two convolutional layers and two fully connected layers. The results show that out of the total of 960 tasks (60% of which marked “bradykinesia present”), approximately 91% were classified correctly.

ML algorithms require a set of features as the input to predict/classify an outcome. Dependent on the type of input and the choice of algorithm, different techniques may be used to represent the input parameters. When the input data is derived from wearable sensors, typically signal processing approaches, possibly combined with sliding windows, are used to extract features (Dinesh et al., 2016). Recent studies have started exploring the use of raw data in CNN to allow the algorithm find the best way of extracting features from the input (Eskofier et al., 2013). Sometimes, the use of all features may result in noise or inaccuracies in the model. In these cases, techniques such as Principal Component Analysis (PCA) may be used to construct linearly uncorrelated variables from the feature set. For instance, Muniz et al. (2010) use PCA to extract features from walking trials data of DBS patients with and without PD. Specifically, they collect ground reaction force (GRF) using force platforms from 43 subjects for 10-s, resulting in 202 GRF samples per subject for both right and left limbs. After preprocessing, they apply PCA to the covariance matrices of sizes 202 × 202 then use the broken stick criterion (Jolliffe, 2002) to determine the number of principal components to retain in the model. Finally, the authors use the resulting extracted features in various ML algorithms including probabilistic neural network, LR and SVM to stratify PD and non-PD patients. These models achieved average accuracies of approximately 93, 92, and 95%, respectively.

## Unsupervised learning

As discussed, apart from the aforementioned machine learning algorithms, which generally fall or are typically used for supervised learning (learning when there is a ground truth/information from direct observations), there are some algorithms that are mostly developed for unsupervised learning or clustering. These algorithms are generally used for exploratory data analysis. For instance, K-means algorithm aims to cluster the observations into K pre-defined number of groups based on their similarities. Palmerini et al. (2013) applied K-means to identify clinical subtypes of PD based on the data from a postural test. They assess the clustering results using Silhouette value (Rousseeuw, 1987), which is a measure of the dissimilarity of each subject with other subjects within the cluster and across other clusters. They results indicate that their clustering structure obtains an average silhouette value of 0.7, suggesting a reasonable-to-strong structure captured in the data. In another study (Hssayeni et al., 2016) a semi-supervised classification algorithm based on K-means was developed to automatically assess the ON and OFF medication states of PD patients based on the motor fluctuations recorded by the sensors worn on the trunk as well as the leg on the side that is more affected by PD motor impairment. In this semi-supervised algorithm, first clustering is performed to group data based on similarities and then two approaches are used to assign ON and OFF medication states labels to the clusters. The authors use the data collected from triaxial gyroscope sensors for 12 PD patients engaging in a set of activities, such as drinking from a cup, walking, and cutting food, and derive features such as the rate of change in the angular velocity, signal power, entropy, correlation coefficient, among others. The results indicate an average accuracy of 76% for all patients, ranging from approximately 43 to 100% across individual patients.

## Challenges in machine learning platform applications

Machine learning algorithms vary from many aspects such as the inherent rationale, effort needed for training, and the number of hyperparameters to tune with respect to the noise level of the data collected, etc. Overfitting or underfitting can occur in statistical learning (Friedman et al., 2001). Overfitting occurs when the model excessively adapts itself to the training data to the extent that it no longer generalizes well to additional/future data. On the other hand, underfitting occurs when the model is missing important parameters or terms, hence it cannot sufficiently capture the relationship between observations and response variables, resulting in poor performance and generalization.

In general, unless the model is underfitted, given an appropriate hypothesis, a large dataset results in better performance from algorithms. Certain algorithms, such as DT and RF, generally have few hyperparameters and hence, can perform well using relatively small datasets. However, some algorithms, i.e., NNs, typically *require* large amounts of data and high efforts for training to perform reasonably well, mainly because of the generally high number of hyperparameters that need to be tuned. Because of the high number of hyperparameters, NNs are generally prone to overfitting. Hence, it is important to use an array of techniques (e.g., regularization, cross-validation, etc.) (Friedman et al., 2001), to avoid such issues.

When the properties of the data sets change over time (non-stationary data), the drifting latent dependencies create a serious challenge in traditional ML. ML algorithms are generally trained on curated data sets collected under specific conditions. In the PD population, the dynamic non-stationary nature of their symptoms will likely lead to a discrepancy between the trained and observed data. While simpler algorithms may allay some of the data drift issues, its predictive value is low due to underfitted models (Kubota et al., 2016). Hence, harnessing large training data from a diverse set of patients over various sensor deployments would be preferred mechanism of implementation for this study population. However, robustness for feedback application may be limited as a result (Kubota et al., 2016). In addition, online machine learning methods have been successfully used to deal with non-stationarity (Bifet, 2010; Calandra et al., 2012).

Since machine learning algorithms are not modular, it is imperative that the input data be in the same form as the trained data (Sculley et al., 2015). This must be taken into account when considering the type of inertial sensor and ML systems to use in a study design. Furthermore the interpretability of the data from PD cohorts have been fraught with misleading information including actions that mimic tremor or lack of environmental changes to analyzing gait in a real world environment (Michael, 2016).

Therefore, while correlating ML models with clinometric scales such as the UPDRS may have research relevance, there may be limited translational use for this approach due to the continuous and multidimensional data that is acquired from wearable sensors.

## Conclusion

The rapid growth of wearable motion sensors in assessing PD symptoms offers new clinical insights into the nature and characteristics of motor disability. There remain considerable variability and lack of standardization in the technology platforms, type of clinometric data acquired, and remote monitoring resolution as it relates to sensor location(s). Nevertheless, the objective, non-biased data provided by wearables not only stands to augment clinical care, but also engenders an opportunity to deliver a more individualized treatment approach to a disease that has phenotypic and genotypic heterogeneity. With closed-loop DBS and new drug delivery pump systems on the horizon for PD, one can envision sensor data acting as a feed-forward mechanism to refine and modulate the degree of therapeutic gain. Such real-time measurements can also serve as the foundation for developing predictive models and stratification of patients into various treatment modes enabling more efficient management of their motor disability.

## Author contributions

RR, AK, and OS designed and prepared the manuscript. BK provided important intellectual input as well as reviewed and critiqued the manuscript.

### Conflict of interest statement

The authors declare that the research was conducted in the absence of any commercial or financial relationships that could be construed as a potential conflict of interest.
